# Agricultural innovations for sustainability? Diverse pathways and plural perspectives on rice seeds in Odisha, India

**DOI:** 10.1007/s10460-024-10666-0

**Published:** 2024-12-04

**Authors:** Saurabh Arora, Bhuvana Narayanarao, Nimisha Mittal, Rasheed Sulaiman Vadekkal

**Affiliations:** 1 University of Sussex, Falmer, Brighton, BN1 9SL UK; 2 Centre for Research On Innovation and Science Policy (CRISP), Hyderabad, India

**Keywords:** Sustainable development goals (SDGs), Science Technology and Innovation (STI), Transformation, Seeds, Rice, India

## Abstract

We focus on *alternative innovation pathways* for addressing agricultural sustainability challenges in Odisha, India. The first pathway that we term as industrial, is focused on breeding new seed varieties in modern laboratories and test fields, ostensibly for climate resilience. It is driven by public scientific institutions and private corporations. The second pathway that we call agroecological, is grounded in saving and sharing of diverse local varieties, largely by Indigenous (Adivasi) smallholders and their allies in civil society. Using the pathways’ descriptions as bases, we present perspectives of different professional groups who appraise how effectively each pathway addresses a range of sustainability issues. While all participants appraise the agroecological pathway to be clearly better performing for addressing agricultural biodiversity and cultural uses of rice, appraisals for issues of the economy, seed accessibility, stress tolerance, and nutrition diverged from each other. An overall picture in support of one pathway did not emerge. Embracing such ambiguities and uncertainties associated with appraisals, we argue for balancing political support between diverse pathways. Greater support for structurally marginalised agroecological pathways may be crucial to meet sustainability goals. This support can include the restitution of lands and other socio-ecological resources for marginalised pathways as well as guaranteeing autonomy of Adivasi (Indigenous) communities among whom the pathways thrive.

## Introduction

Sciences, Technologies and Innovations (STIs) are widely seen as central to addressing agricultural sustainability challenges, including those highlighted in the Sustainable Development Goals (SDGs) (UNIDO and UNIATT 2022; Senise et al. 2021; Matusiak et al. 2021; Adenle et al. 2020; Lee and Mathew 2013). However, globally dominant STIs are considered strongly misaligned with many sustainability priorities (Ciarli et al. 2022; Antwi-Agyei et al. 2018). Beyond transforming dominant STIs, it is therefore crucial to foreground marginalised pathways of STI development.

Given the global hegemony of modern STIs and the extensive scope of SDG priorities, alternative STI pathways in specific areas are often obscured or treated as unviable (see e.g., Jansen 2015). To illuminate alternative STI pathways (Leach et al. 2010), we focus on a particular agrarian context – rice seeds in Odisha, India—and present comparative appraisals of different STI pathways’ misalignments with SDG priorities. The appraisals are not aimed at closing down to establish the overall superiority of one pathway, but rather at opening up the politics of STI-SDG alignments by highlighting uncertainty and ambiguity associated with actors’ plural perspectives (Stirling 2008). We illustrate how the perspectives diverge or converge, and taken together what they might mean for wider agricultural sustainability.

Even though multiple directions of STI development can be found in any area of activity (e.g., agroforestry, organic cultivation, and modern industrial agriculture), power closes down this multiplicity of STI pathways. It is thus typically the case, that dominance is gained by just one (or few) STI pathway(s) (Stirling 2009; Arora et al. 2019). This dominance may be realised and maintained through processes such as: (i) prevalent methodologies that privilege particular performance criteria (eg: crop yields per hectare) that make one STI pathway appear significantly more viable and effective than alternatives (Vanloqueren and Baret 2009); (ii) technological lock-in and the accumulation of interests around a locked-in direction (Unruh 2002); (iii) material obduracy of established infrastructures which limits possibilities for transformation (Hommels 2005); (iv) prevalence of policymaking routines that suppress intractable uncertainties in favour of probabilistic risk (Callon et al. 2009); and (v) discourses of development that reduce progress to economic growth while marginalising wider socio-ecological sustainability, despite rhetorically deploying the SDGs or other greenwashing (Kothari et al. 2019). To counter these manifestations of power that close down alternative STI pathways, concepts and methods for opening up are required (Stirling 2008). Crucial among these are methods for pluralist appraisals of diverse pathways, which attend equally to perspectives of marginalised actors alongside those of privileged experts, regulators and investors.

We describe two contrasting STI pathways for rice seeds in Odisha: (1) an industrial pathway focused on developing new varieties of rice in ‘controlled’ environments of modern laboratories and test fields; and (2) an agroecological pathway structured around varieties developed, conserved and shared primarily by Odisha’s Adivasi (or Indigenous) smallholders. Using the pathways’ descriptions, we conducted appraisals of STI-SDG misalignments using the qualitative-quantitative method of multi-criteria mapping (MCM) with a diverse group of participants (Stirling and Coburn 2014). MCM helps highlight differences and ambiguities among participants’ plural perspectives, rather than integrating them based on ‘representative samples’ to derive general conclusions and implications. This does not mean that possibilities for consensus among participants are foreclosed: consensus is emergent where participants’ plural perspectives are in agreement with each other.

MCM allows each participant to highlight the uncertainty associated with their appraisals. In addition, in appraising different pathways for meeting a focal goal, participants use criteria that are self-defined by them. We urged participants to define those criteria for appraisal, which they consider as relevant to the SDGs. Collectively the participants defined 68 criteria, which we grouped into seven issues.

For issues of biodiversity conservation and for usability (that includes taste, fragrance and other cultural values attached with specific rice varieties), participants favour the agroecological pathway. For most other issues such as those related to the economy, plant stress and accessibility (of seeds), there is no clear-cut consensus. Embracing this ambiguity and divergence among plural perspectives, we call for strengthening the equal coexistence of both appraised pathways. We conclude therefore that the currently neglected and marginalised agroecological pathway for seed development in Odisha (and other parts of India), be protected from further damage by agricultural modernisation (rather than co-opting agroecologies into modern nationalist and capitalist projects, see eg: Fitzpatrick et al. 2022; Ramdas and Pimbert 2024). For this, we call for greater political autonomy of Adivasi farming communities over lands and seeds.

The paper is organised as follows. In the following section, we offer an overview of the literature on misalignments between agricultural STI pathways and SDG priorities, focusing on India. Subsequently, we discuss the conceptual framework and the methods used in this study. We include a brief discussion on the dynamics of the two pathways and how they are constituted, including reasons why the industrial pathway remains dominant. Issues related to alignment of pathways to SDGs based on MCM results are then analysed, which is followed by a final discussion and conclusions section focused on ways to address STI-SDG misalignments.

### Literature on (mis)alignments between STIs and SDGs

It is widely believed in policy circles and wider society that modern STIs are central to achieving sustainability goals. In general, however, the goals are understood as technical targets and therefore apolitical and uncontested, while modern STIs are considered as politically neutral and universally applicable (Ajwang et al. 2023; de Hoop and Arora 2021). Such a solutionist approach to sustainability using modern STIs (Morozow 2014; Asafu-Adiave et al. 2015), tends to overlook that there are multiple possible pathways of STI development (Stirling 2009), some of which (like those based on toxic pesticides, fossil fuel extractions and opencast mining) may be directly linked to exacerbating sustainability challenges. In this context, recent literature on innovation has begun to highlight that currently dominant patterns of STI development are poorly aligned with SDG priorities (Ciarli et al. 2022; Manninen 2022).

Such analyses of STI-SDG alignment are made challenging due to political interconnections of different SDGs with each other (Aminullah 2020). While individual SDGs neatly separate socioeconomic imperatives from environmental targets, it is difficult to enact the same separation in practice for STIs. The use of particular STIs like chemical pesticides and genetically modified seeds, ostensibly for addressing a socioeconomic imperative such as poverty or hunger (SDG 1 or 2), may be associated with adverse consequences for life on land (SDG 15) and often also for climate change (SDG 13). For instance, wind energy developments for addressing SDG 13 (focused on climate action), can have adverse socio-economic and cultural consequences for communities on whose lands the wind turbines may be located (Avila-Calero 2017)—lands that may otherwise be the bases for sustaining livelihoods in agriculture and pastoralism (directly tied with SDGs 1, 2, and 3 focused on poverty, hunger and health and wellbeing). Therefore, in STI actions and investments, the three oft-invoked pillars (economic, social and environment) of sustainability are politically entangled with each other. The foregoing also means that even where specific STIs may positively contribute to addressing some SDGs, they can at the same time be seriously misaligned with other SDG priorities.

In attempting to map such misalignments at the country level, recent studies find that research fields accounting for most funding and publications in a country, are not the ones in which the country faced the most challenges in achieving the SDGs (Confraria and Ciarli 2022; Ciarli and Rafols 2019). For example, in relation to rice, human nutrition is not a focus of research in countries where rice is widely consumed as a staple and where malnutrition is considered to be high, but rather nutrition-focused research is carried out in countries that are large exporters of rice (Ciarli and Rafols 2019).

It may be argued that such misalignments are outcomes of poorly considered national policy agendas for STI development. Among others, modern policymaking may privilege export incomes over domestic needs (particularly needs of impoverished people); and they may promote technocratic industrialisation over socio-ecological wellbeing grounded in plural ways of knowing (de Hoop and Arora 2021; Arora et al. 2020). Policies may also reduce questions of health to modern medical cures while marginalising nutritional care (Kumar 2019; Glover and Poole 2019).

While these critical views on national (and regional) policies are insightful, it is crucial to situate national and regional contexts in wider transnational flows that materialise either through pressures to catch-up with ‘*the* global’ frontier in modern STIs (eg: to win the so-called ‘arms race’ in advanced technology) (Asaro 2019), or through national/regional policies’ cascading effects on other connected regions (Downing et al. 2021). An example of the latter is China’s reforestation programme having adverse impacts on neighbouring countries that export plantation rubber, timber, soy, and palm oil to China. Exporting countries suffer deforestation and pollution, which compromises their ability to meet SDGs 6 (clean water), 13 (climate action), 14 (life below water) and 15 (life on land). Any benefits for SDGs focused on socio-economic imperatives, were observed to be unequally distributed in exporting countries – governed by structural social inequities (UN CDP 2013).

Development of modern STIs for rice production—an important staple crop for over three billion people globally (Yadav and Kumar 2019)—has received significant policy and funding attention in the last 6–7 decades since the green revolution (GR) (Cullather 2004; Pingali 2012). In India for example, STIs such as semidwarf high-yielding varieties and chemical fertilizers and pesticides (Mukherji 2022; Yadav and Kumar 2019), are celebrated for affording a five-fold increase in rice production (from 20 million tonnes in 1950–51 to 110 million tonnes by 2018–19) and more than two-fold increase in yields (from 1.5 tons/ha to 3.8 tons/ha during the same period) (GoI 2019). These figures support claims that modern industrial STIs in agriculture are well-aligned with SDGs 1 and 2 (poverty and hunger). Where such a selectively positive reading of modern STIs in agriculture is privileged (Kumar 2019; Arora and van Dyck 2021), they may be presented as generally aligned with the SDGs. However, any claim of general alignment overlooks many adversities associated with such STIs.

For example, in rice production, modern STI developments are associated with environmental degradation, particularly of soil, biodiverse vegetation and water resources (Singh 2000). Promotion of rice-based diets over many different kinds of millets is linked with protein malnutrition and nutrient deprivation (Shatrugna 2010). The introduction of modern rice varieties and practices of monoculture has led to reduction in and loss of agricultural biodiversity (Nelson et al. 2019; Cabral et al. 2021). Additional challenges are associated with exacerbating climate disruptions, which intersect and overlap with depleting groundwater tables, water salinity and alkalinity problems, deteriorating soil qualities, and frequent floods leading to complete submergence of rice plants, erratic rainfall and seasonal patterns (Singh 2000; Dar et al 2017; Singh et al 2017).

Overall then, the promotion of modern industrial STIs aimed at increasing agricultural production, tends to overlook complex relations between socioeconomic development and environmental change. Under selective readings, industrial STIs in agriculture may be promoted and celebrated for contributing to (socioeconomic or climate) priorities of one or two SDGs (Nerini et al. 2019), to neglect how they may be misaligned with a wider range of SDG priorities.

Beyond the SDGs per se, central here is the agrarian distress unfolding in India over the last three decades, which is associated with high costs of modern farm-inputs, widespread suicides by indebted farmers, and rampant environmental depletion (Vasavi 2012). For addressing this crisis, it is often emphasised that modern patterns of STI development associated with the GR must be transformed (Arora 2012; Shankari 2018; Khadse et al. 2018; Cabral et al. 2021). STIs for rice are crucial to this transformation. There are at least two interrelated ways in which such transformations are approached:

- *Extending the adoption of modern STIs among smallholders*: Firstly this involves improving small and marginal farmers’ *access* to modern STIs like hybrid (or genetically modified) seeds and synthetic fertilisers, the development of which may be driven by formal agricultural research institutions and corporations (Spielman et al. 2013). In a broader frame, focus may be on learning by STI institutions and systems by directing attention to farmers’ diverse experiences of adopting and using STIs on the ground (Hall et al. 2014). It is here that farmers’ creative adaptations of modern STIs may sometimes be institutionally embraced (Jones-Garcia and Krishna 2021).
- *Promoting the diversification of STI directions*: Transformations to sustainability may be based on the recognition that there exist STIs beyond those developed by modern institutions like publicly funded laboratories and private corporations. Alternative STIs may be based on the development and circulation of smallholders’ knowledges and innovations, to constitute pathways like agroecology, agroforestry, organic cultivation and ‘zero budget natural farming’ (Arora et al. 2013; Khadse et al. 2018). Similar innovative efforts by civil society organisations may also sometimes be considered significant (Prasad 2020). Crucial here are attempts to challenge entrenched power relations that close down STI directions to modern industrial agriculture, and thereby marginalise alternatives (Stirling 2008; Ajwang et al. 2023).

Often central in closing down alternative directions of STI, is the power of a few large transnational corporations (TNCs) developing modern STIs like chemical pesticides and genetically modified seeds (Jasanoff 2006; Gliessman 2023). Working with national/regional governments and local businesses, such corporations can aggressively promote their STIs at the expense of alternatives such as historical varieties that are saved and exchanged between (smallholder) farms. The higher costs of TNCs’ STI-based farm inputs, particularly patented seeds, are also considered to be an important factor behind indebtedness of smallholders and therefore wider agrarian distress (Vasavi 2012; Gupta 2017).

To challenge corporate consolidation, agroecological food and seed sovereignty movements such as La Via Campesina internationally and Beej Bachao Andolan in India (Moudgil 2017; Pimbert 2022), are crucial for protecting and nurturing alternative STIs for agricultural sustainability. Equally important in India, is the seed sovereignty work of civil society organisations like Navdanya and Deccan Development Society, which support community seed banks and participatory breeding (Cruz and Fliert 2023). Few such movements, however, are free from the influence of rural elites, large corporations, government institutions and transnational civil society (Brown 2018). Where co-opted by nationalist agendas or big capital (Ramdas and Pimbert 2024; Fitzpatrick et al. 2022), movements can extend modernisation of agroecologies rather than offering viable alternatives to agricultural modernisation pathways.

The experience of transnational developments in agroecology are instructive in this regard. As a term agroecology is traced back to the 1930s. Claimed to be used exclusively within modern discourse till the 1960s, agroecology ensues as a practice only in the 1980s and as a social movement since the 1990s (Wezel et al. 2009; also see Toledo and Argueta 2024 for a country study on topic). Such an ‘origin story’ tends to prioritise the academic term agroecology – situated within a modern scientific discourse dominated by the Global North (Gómez et al. 2013) – over farmers’ practices and movements in different parts of the world (eg: Rosset and Martínez-Torres 2012). Focusing on the latter practices and movements as distinct from modern industrial agriculture that is seen as emerging in 1850s Europe (eg: Moser and Varley 2013), agroecology thrives in many different forms developed by diverse (vernacular and Indigenous) ways of living and knowing over centuries and millennia (eg: Garí 2001; Altieri 2002; Ganz 2005). It is such practices developed by Adivasi smallholders and their allies in an Indian region, which we approach as *agroecological* in the following.

The literatures reviewed above offer valuable insights on (asymmetric connections between) dominant and marginalised agricultural STI pathways with respect to sustainability transformations. However, studies on possible coexistence and multi-criteria comparisons of diverse STI pathways are rare in the literature. We address this neglect by analysing two contrasting pathways of developing rice seeds while comparing their (mis)alignments with priorities of the SDGs for a range of criteria defined by our study’s participants. The first pathway that we term *industrial*, is focused on modern scientific breeding and industrial production of high-yielding rice varieties claimed to tolerate stresses and build climate resilience. The second pathway is focused on in situ conservation and development of diverse local varieties of rice that may also be considered stress-tolerant, which we refer to as the *agroecological pathway*.

## Concepts

Growing recognition of sustainability challenges and their drivers in modern STIs like industrial chemicals and fossil fuel extraction (e.g., Unruh 2002; Sánchez-Bayo and Wyckhuys 2019), means that greater attention is being given not only to modern greening agendas like the sustainable development goals (SDGs) but also to alternative possible directions of STI development (Stirling 2009; Arora et al. 2019). To support alternatives, it is argued that a shift is required in STI policy and politics through which power closes down the multiplicity of STI directions (Stirling 2008; 2009; Schot and Steinmuller 2018). In agriculture power relations since the colonial era, have meant that postcolonial development policy has almost exclusively promoted modern industrial STIs like toxic pesticides, synthetic fertilizers and seeds developed in laboratories (Ajwang et al. 2023). Modern industrial agriculture’s success in increasing crop yields per unit of land and labour, has been glossed as the green revolution (GR) in many parts of the world (Cabral et al. 2021). While new GRs are sought by powerful funders like the Gates Foundation (AGRA 2013), multiple STI directions grounded in alternatives like agroecology and agroforestry remain margnalised in public policy for development and sustainability in postcolonial regions (Lappe 2016; Brown 2018; Ajwang et al. 2023).

To help direct greater attention to alternative STI directions, several conceptual approaches are relevant. Consider for example sustainability transitions frameworks in which societies may be seen as evolving at multiple levels. Here distinctions are made between socio-technical regimes structured around dominant incumbent forces and lower-level niches in which potentially radical innovations are protected and nurtured (Geels 2002; Smith and Raven 2012). Through further development, innovations from niches are seen as contributing to a regime shift, or *the transition* imagined as leading to a more ‘sustainable’ socio-technical system (for production and consumption) in a specific area of activity like agriculture or energy (Cohen 2021; Arora and Stirling 2023). However, sustainability transitions frameworks are marred by two fallacies: a) use of *reifying concepts* like niche and regime that not only approach reality as hierarchically ordered (in levels and systems), but also concretely equate the concepts to reality itself (Stirling 2018); b) *depoliticisation of inequalities* in socio-ecological consequences of emerging innovation pathways and the colonial modern world they help make, across the Global North and South (Arora and Stirling 2023).

In this way, by approaching sustainability as a natural transition from one dominant system or regime to another, such frameworks do not offer insights into how multiple neglected directions of STI for sustainability can be highlighted and supported together. To address the above concerns, we ground our analysis in the pluralist *pathways approach* (Leach et al. 2010). Fundamental to this approach are *framings of sustainability problems*, because different problem framings direct attention to contrasting STI pathways. The latter pathways are constituted by STI actors, practices and relations.

We approach STI pathways not as actually existing reality manifesting for example as practices of farmers, scientists or other actors. As concepts pathways highlight diverse possible directions of STI development (and new pathways can always be developed from combinations of existing ones). Different pathways may even coexist – in asymmetric relations – within practices of individual farmers (eg: Gupta 1998; Arora 2012). The prevalence of specific STI pathways rather than others, is grasped as an outcome of sociopolitical choices and struggles between contending forces. Furthermore, because different STIs relate with marginalised people and the environment in contrasting ways – for example ranging from categorical ambitions of control and domination to relational values of care and conviviality in practice (Arora et al. 2020), not all pathways are equally desirable when it comes to politics of sustainability considered broadly as it is in the SDGs.

Our pathways approach highlights a pervasive tendency in modern societies characterised by concentrated and accumulated power and privilege (Arora and Stirling 2023), that professional, institutional and political power *closes down* diverse directions of STI development (Stirling 2008). Closing down often ends up favouring particular pathways structured by modern imaginations of control and domination, such as those associated with industrial agriculture (Stirling 2014; Arora and van Dyck 2021). In order to help *open up* alternative STI pathways that may favour marginalised people, or those that are grounded in an ethos of care (Arora et al. 2020), it is important to *broaden out* appraisals of contrasting pathways using a wider range of sustainability criteria (Stirling 2008). Here, broadening out implies that multiple contested “sustainabilities” may be defined and deliberated for any particular issue (like a SDG target), where different groups may offer contrasting yet equally valid *plural perspectives* on the ‘same’ pathway (Leach et al. 2010). Each of the plural perspectives is uncertain. Considered together the perspectives may yield an ambiguous picture regarding overall (mis)alignment between a STI pathway and the SDGs (Ciarli et al. 2022).

Rather than reducing uncertainty and ambiguity to risk that can be calculated and avoided (Callon et al. 2009), the pathways approach embraces them as unavoidable features of knowledge (Stirling 2015). Uncertainties and ambiguities may be suppressed by power to give the impression that policies are resting on authoritatively complete knowledge that has taken risk into account (which helps also to close down alternative STI pathways). By admitting uncertainties and ambiguities in characterising and appraising pathways, as we attempt below, the equal validity of marginalised actors’ perspectives and of vernacular or ‘traditional’ knowledges can be more easily appreciated (Arora 2019; de Hoop and Arora 2021). Such appreciations can in turn nurture the parallel development of *diverse pathways* grounded in multiple ways of knowing – within and beyond individual smallholders’ practices. Embracing diverse pathways may be crucial not only for transforming modern industrial agriculture, but also for supporting pluriversal lifeways that go beyond the universalism of modern STIs and narrowly defined targets constituting the SDGs (Kothari et al. 2019; Arora and Stirling 2020; Arora-Jonsson 2023).

## Methodology

Using a case study research design, we aim to develop detailed insights into the plural perspectives of people performing different roles in the two focal pathways (industrial and agroecological). In particular, the plural perspectives show how the STI pathways are (mis)aligned with the priorities of the SDGs (Ciarli et al. 2022).

Odisha was selected as focal region due to the concerted efforts there of Adivasi smallholders and other ‘seed champions’ in protecting agri-biodiversity. In contrast, research institutions of the state and private corporations promote the dominant pathway developing lab-bred varieties of rice (in Odisha like in most other parts of India). To describe the two pathways, we started by examining a wide range of documents including annual reports of relevant organisations and project completion reports. We then conducted semi-structured interviews with key stakeholders (including three individual ‘seed champions’ and representatives of three locally active NGOs) associated with the agroecological and industrial pathways. Focus group discussions with farmers in 12 villages and a women’s federation were also conducted in Odisha, to understand their work on the conservation and use of rice seeds.

Using the two pathways’ *descriptions* as bases, we used a method called multi-criteria mapping (MCM) (Coburn et al. 2019), to gather plural contrasting perspectives on each pathway’s misalignments with the SDGs. MCM employs a software tool to map a complex problem from different points of view. The aim is *not* to develop a single view that is representative of a group of actors, but rather to highlight a plurality of views across different groups (which may or may not converge). Central to MCM are values of *opening up* pathways through balanced attention to contending views, *inclusion* of marginalised perspectives, *agency* of study participants in defining the issues they find important in apprasing pathways, and *transparency* through conveying results to all concerned parties (Coburn et al. 2019). MCM is thus not a priori programmed towards closing down of pathways. Contrasting methods such as ‘best–worst scaling’ enable comparative appraisals that aim to close down through optimisation that is ostensibly representative of a population, by asking participants to select the *most* and the *least* appealing options (and issues) out of an available set (eg: Finn and Louviere 1992; Louviere et al. 2015).

MCM is a hybrid method highlighting connections between qualitative and quantitative data. Our study’s participants were purposively selected to be able to appraise specific STI pathways as two options for addressing SDG challenges that they deemed as relevant and important. All participants were requested to pay careful attention to the pathways’ descriptions (rather than their titles). Each participant was asked to assign quantitative scores to a pathway’s potential alignment with different SDG priorities, while also providing less tangible qualitative information about the conditions under which they expect their quantitative assessments to be valid. A main aim of the MCM exercise was to broaden out the scope of STI pathways’ appraisals, beyond policymakers and scientific experts. Therefore, we conducted MCM interviews with 20 individuals including farmers and representatives of civil society organizations. Each interview entailed the following steps:

- At the start of each interview, we shared a succinct description of the two STI pathways being appraised.
- We requested each participant to appraise the two pathways based on the sustainability criteria that they themselves defined and considered important. We linked these criteria to the SDGs later during data analysis.
- As no appraisal can be completely certain (Coburn et al. 2019), we asked every participant for two scores – an optimistic and a pessimistic – that they estimated as characterising the performance of a pathway in relation to each criterion defined by them. An optimistic score says how well a participant expected a pathway to address a particular criterion, under conditions they deemed favourable (Coburn et al. 2019). In contrast, a pessimistic score tells us how the participant expected a pathway to perform under conditions that are unfavourable. Crucially connecting with these quantitative scores, we asked each participant to provide qualitative information, to describe what they considered to be the particular unfavourable and favourable conditions.
- The final step of each interview involved reviews by each participant of the appraisals generated by them. If a participant was dissatisfied by their appraisal, we asked them to revisit the scores and revise them as needed.

As part of our analysis using MCM, the difference between pessimistic and optimistic scores provided by a participant, were used to estimate the uncertainty associated with the participant’s appraisal. We relied on the participants’ professional backgrounds to group them into four different perspectives – of policymakers, extension workers, researchers, and farmers. These plural perspectives on each of the two pathways helped highlight points of disagreement and agreement between different partipants’ appraisals. In the MCM interviews, our study’s participants collectively defined 68 criteria. We grouped these criteria into seven substantive issues relevant to the SDGs.

### Diverse STI pathways for rice seeds in Odisha

Rice (Oryza Sativa L) has been cultivated in India for at least 7000 years, adapted to grow below sea level and at high altitudes. Rice is grown in a variety of ecosystems including rainfed uplands and lowlands, tidal wetlands, and canal-irrigated areas (Panda and Pathak 2019). Adapted to this wide range of agro-ecosystems, India has had rich genetic diversity and variability in rice. Historically, farmers have played central roles in generating and conserving rice biodiversity, by experimenting with and sharing seeds (Shiva 1991). Based on agricultural knowledges accumulated over generations, farmers have produced and conserved thousands of local varieties for their potential to tolerate flood, drought and salinity. Many rice varieties also serve medicinal, nutritional, specialist culinary, cattle feed, and other cultural purposes.

However, the biodiversity of rice in India has rapidly declined in the last six decades since the unleashing of the country’s so-called Green Revolution (GR). In this GR, philanthropists joined hands with international agricultural research organizations, development aid agencies, Indian governmental organisations and private corporations to promote industrial agriculture involving toxic pesticides, synthetic fertilizers and ‘high-yielding’ varieties of seeds (eg: Farmer 1977). It is estimated that before the GR, more than 100,000 varieties of rice were grown in India (Nelson et al. 2019). This figure is now considered to be lower than 7000. By the 1990s, the bulk of India’s rice production was focused on less than 50 modern industrial varieties. Below, we describe one STI pathway based on modern scientific breeding of seeds associated with industrial agriculture, and an agroecological pathway that focuses on conserving diverse local varieties of rice in Odisha, India (Ciarli et al. 2022). Following these descriptions, we present our comparative analysis of the two pathways’ (mis)alignments with the SDGs.

### Industrial pathway for rice seeds

Frequent droughts, floods and cyclones, impact agricultural production. Attempting to tackle such shocks and stresses that are often associated with climate change, agricultural scientists breed ‘improved’ rice seeds designed to be high-yielding. Most seed breeders are based either at Odisha University of Agriculture and Technology (OUAT) or at the national government’s Indian Council of Agricultural Research (ICAR) that includes the National Rice Research Institute (NRRI) in Cuttack, Odisha. Between them, NRRI and OUAT have disseminated roughly 200 seed varieties that are adapted to four main rice ecosystems in Odisha – rainfed-lowlands, rainfed-uplands, flood-prone areas and irrigated lands.

Beyond NRRI and OUAT, our document mapping and semi-structured interviews (with key stakeholders) highlighted the roles of many other actors in the industrial seeds pathway. These actors and their roles are listed in Table 1. While government institutions dominate, crucial roles are played by private companies in selling (and diffusing) lab-developed seeds. The role of smallholders in this industrial pathway is largely limited to use. They buy seeds bred elsewhere, although many also cultivate their own farm-saved seeds and exchange seeds with other farmers.

**Table 1:** Actors in Odisha’s industrial pathway

Actors	Central roles
*Indian Council of Agricultural Research* (ICAR)	• Provides financial and administrative support for seed development; • Supports AICRIP (All India Coordinated Rice Improvement Programme) to lead trials of lab-developed seeds
*ICAR-National Rice Research Institute* (NRRI), Odisha	• Involved in research, development and dissemination of high-yielding varieties (HYVs); • Works collaboratively with local Krishi Vigyan Kendras (see below), NGOs and farmer-producer companies to organise demonstrations of new seed varieties’ potential; • Collects rice germplasm
*National Seeds Corporation*	• Involved in the supply of rice seeds, particularly for National Food Security programme (and other similar programmes); • Organises to bring Green Revolution-style missions to eastern India;
*Krishi Vigyan Kendras* (district centres set up by ICAR to provide farm support)	• Organises on-farm trials of new varieties; • Organises demonstrations and conducts training to promote new HYVs
*Odisha state Department of Agriculture & Farmers’ Empowerment*	• Involved in the promotion of HYVs and hybrid varieties as recommended by OUAT; • Responsible for implementing government programmes for promoting new seed varieties, also by providing subsidies and organising demonstrations
*State Seed Testing Laboratory* (SSTL), Government of Odisha	• Performs tests on seed samples for controlling quality; • Organises for the validation and certification of rice seed varieties assembled from different regions of Odisha; • Organises training in seed testing technology, provided particularly to seed growers and traders
*Odisha state seed Corporation* (OSSC)	• Certifies seeds produced through programmes that involve growers; • Coordinates the sale of certified seeds through government-run and private shops
*Odisha State Agro-Industries Corporation*	• Regional government agency responsible for helping to bring seeds to market
*Odisha University of Agriculture and Technology* (OUAT)	• Does research, development and dissemination of HYVs • Helps evaluate new seed varieties by coordinating a local AICRIP centre for Odisha; • Responsible for hosting and managing a regional station for research and technology transfer
*International Rice Research Institute* (IRRI), the Philippines	• Organises for access to global ‘elite’ rice germplasm; • Organises training of Indian scientists in ‘advanced’ rice breeding methods
*IRRI Odisha State Office*	• Helps promote ‘climate-resilient’ rice varieties in collaboration with OSSC, Department of Agriculture and NGOs, often by organising, cluster demonstrations, field-level evaluation, training and other capacity development
*Seed growers* (about 5000 registered with OSSC) and *Seed producer groups*	• Cultivation of seeds, to be validated, certified and labelled (all of which is facilitated by SSTL)
*Private sector seed companies*	• Selling of roughly 5,000 metric tons of rice seeds each year in Odisha

### Agrocological pathway for rice seeds

The alternative STI pathway promotes rice seeds that are grown, saved and shared primarily by Odisha’s Adivasi peoples and some local farmer-activists. This pathway is thus structured mainly around *in-situ* development of new varieties – often based on conserving ‘heritage’ or ‘heirloom’ varieties such as *Kolaajeera* and *Patraa-dhaan* – between small farmers. Central to this pathway is the sustaining of seeds and crops in their ecological habitats. In sharp contrast to the industrial pathway’s attempts to centralise research, development, testing and distribution of new varieties, the agroecological pathway may be structured around decentralized Community Seed Banks that help save and share local seeds.

Our focus group discussions revealed that farmers often share seeds freely. The sharing and development of seeds is also facilitated by individual seed champions, seed saving groups, local civil society organizations and community seed banks (see Table 2). Many Adivasi smallholders claimed that they prefer their own seeds for reasons of taste and aroma. Often, these varieties are also important in celebrating cultural festivals and conducting religious rituals. The straw from harvested rice may be used as fodder for cattle and sometimes in the thatching of domestic roofs. It is claimed by Adivasi farmers that their local varieties are also less vulnerable to pest attacks and plant diseases. They may generally use very little, if any, synthetic fertilizers and pesticides in growing local varieties.

**Table 2:** Actors in the agroecological pathway in Odisha

Actors	Central roles
*Civil society organizations* (CSOs) such as Pragati, MS Swaminathan Foundation, and Living Farms	• Involved in documenting agri-cultural and nutritional characteristics of farmer-led (which may be based on heritage or heirloom) rice varieties, while also promoting their cultivation (for selecting seeds that can be saved and shared further); • Seek feedback from farmers and their groups, as well as consult scientists, on varietal performance including stress tolerance, yields and disease resistance; • Organise community-based seed bank and seed exchanges (also through specialised events); • Help provide training, particularly to smallholder farmers, on seed quality and storage
*Individual seed champions*	• Contribute to revive those varieties that are considered to be critically endangered, often by practising organic cultivation on their own lands; • Central roles played in collecting, cultivating, saving and sharing many local varieties, often through collaborative work with CSOs
*Seed growing farmers* (mainly Adivasi) and their groups	• Involved in conserving and growing heritage or heirloom varieties of rice as well as newer ones; • Create networks with other farmers, in which seeds are often freely shared
*State Seed Testing Laboratory* (SSTL), Government of Odisha	• Collection and storage of rice varieties, by sourcing them from (Adivasi) farmers and preparing the seeds for scientific ‘validation’; • Centrally involved in ‘evaluating’ local varieties’ quality characteristics

Our interviewees noted that public and private investments or other support provided to the agroecological pathway has been marginal in comparison to the industrial pathway. Government support has been largely limited to some civil society organizations carrying out seed conservation work as part of larger institutional programmes like the Women Farmers Development Programme. The agroecological pathway remains largely hidden or neglected in the policy debates around increasing rice production, agricultural productivity and sustainability. Overall then, the industrial pathway is seen as dominant for rice seed production in India.

Working with the assumption that both pathways can contribute to improving access to seeds of new rice varieties, in the following we examine how participants in our Multi-Criteria Mapping (MCM) study appraise the pathways’ (mis)alignments with different challenges addressed in the SDGs.

### Plural perspectives on STI pathways and SDGs

Rather than attempting to capture the definitive perspective on STI-SDG misalignments, we aim to appreciate a plurality of perspectives. For this, we identified four different perspectives among the 20 participants interviewed for the MCM study (Ciarli et al. 2022). These perspectives are:

• Farmers (two women and four men growing rice);

• Extension workers (one woman and three men engaged in the promotion of technology among farmers);

• Researchers (five men and one woman focusing on the development of modern technology for rice production);

• Policymakers (one woman and three men with professional capacities in agricultural policymaking).

For their individual appraisals using MCM, we shared with each participant brief descriptions of the two pathways. Based on these descriptions, participants were asked to appraise both pathways using sustainability criteria that they themselves defined and considered as important. Using the 68 criteria collectively defined by the participants, we derived seven *issues* that are closely related to the SDGs (as part of a larger study: Ciarli et al. 2022).

These issues are:

- *Agrobiodiversity* (related to SDGs 15, 13, 3 and 2): biological diversity of rice cultivars used and the conservation of gene pools;
- *Plant stress* (SDGs 2, 13): rice varieties’ tolerance to biotic and abiotic stresses;
- *Accessibility* (SDGs 1, 2, 3, 5, 10, and 12): improved access to farm-inputs and extension services, particularly for farmers from marginalised communities;
- *Economy* (SDGs 1, 2, 8 and 12): security of farmers’ livelihoods (particularly incomes and market value), crop yields and quality as well as national rice production and income;
- *Nutrition* (SDGs 2, 5): nutritional value of rice varieties and their contribution to households’ nutritional security;
- *Usability* (SDGs 3 and 12): flavour, fragrance, consistency and other cultural values associated by users-consumers with rice varieties;
- *Others*: diverse criteria such as plant height, prestige, research accuracy, and trait-specific preferences.

### Farmers’ perspective

**Fig. 1:**
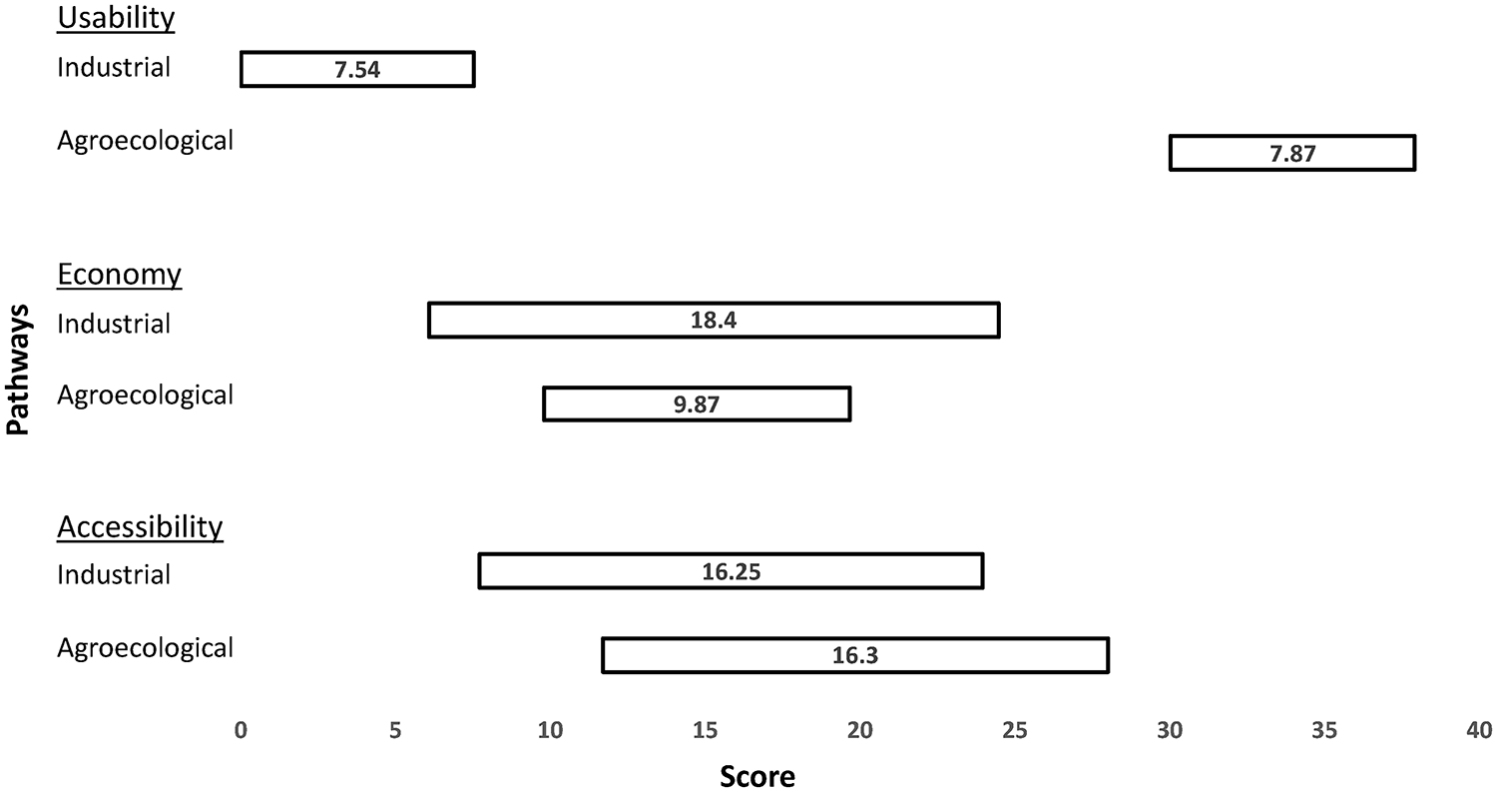
Farmers’ perspective on industrial and agroecological pathways in Odisha, for the three issues of usability, economy and accessibility

Farmers considered the issues of usability, economy and accessibility as salient. Their appraisals of the two STI pathways’ (mis)alignments with SDG challenges are shown in Fig. 1 (where the issues are underlined and uncertainties are the figures inside bars).

#### Usability

For this issue, the average optimistic *and* pessimistic scores attached by farmers to the agroecological pathway are far higher than the industrial pathway. Thus, for culturally-oriented criteria like the rice varieties’ taste, fragrance and uses in rituals, farmers consider the agroecological pathway to be better than the industrial pathway. For this issue, farmers’ appraisals of the two pathways were equally uncertain (see numbers inside the bars in Fig. 1). As compared to the farmers’ appraisals for the other two issues (economy and accessibility), uncertainties are lowest for usability.

#### Economy

In terms of the mean pessimistic scores for the issue of economy, farmers consider the agroecological pathway to be better performing than the industrial pathway. Farmers justify this appraisal by highlighting that the costs of external inputs in the cultivation of local varieties (central to the agroecological pathway) are close to zero, which means that their losses under bad conditions that lead to a poor harvest are more tolerable. In contrast, for cultivating rice in the industrial pathway, farmers need to buy seeds, pesticides, fertilizers, and other external inputs. Under favourable conditions such as an easily accessible and guaranteed market (plus good rainfall) where they can get a good price for their produce, farmers consider the industrial pathway’s high yielding varieties (HYVs) to potentially yield a higher income. This was the reason why the farmers gave a higher mean optimistic score for the industrial pathway. Considering the farmers’ rationales described here, it is unsurprising that they associate a higher uncertainty with the industrial pathway (see the numbers inside bars in Fig. 1). This uncertainty is also documented widely by the literature on India’s agrarian crisis that we reviewed earlier.

#### Accessibility

For accessibility, farmers focus on the availability and costs of seeds. Their appraisals of the two pathways are rather similar (also for uncertainties), although some farmers problematised the HYVs’ higher costs, erratic availability, and the difficulty of saving seeds on farm. The industrial pathway is therefore appraised as performing somewhat worse than the agroecological pathway. According to one farmer:

“I am not sure about the source of availability of HYV seeds. If the seeds are available at the block level, then we have to pay a lot of money to get them. Usually, they say that the HYVs can be stored for two to three years but often we cannot use our saved HYV seeds even in the next year.”

The availability of seeds in the agroecological pathway was also considered problematic by some farmers. Knowledges about producing such seeds were seen as not always readily available in local farming communities these days:

“Farmers often do not have seeds of shorter duration traditional varieties for lowlands, which are resistant to lodging.”

Farmers’ appraisals indicate that institutional support for just one pathway (as is currently the case for the industrial pathway) is unlikely to address the many challenges of the SDGs. Supporting diverse STI pathways appears more fruitful in the farmers’ view. This is not the same as supporting a diversity of modern technologies for seeds, from HYVs to hybrids and genetic modification, which may all be developed within the industrial pathway. Instead, diversity of alternative pathways highlights the need of supporting in situ development and sharing of the varieties developed by farmers, which are innovative activities that flourish largely outside modern agricultural research systems.

### Extension workers’ perspective

**Fig. 2:**
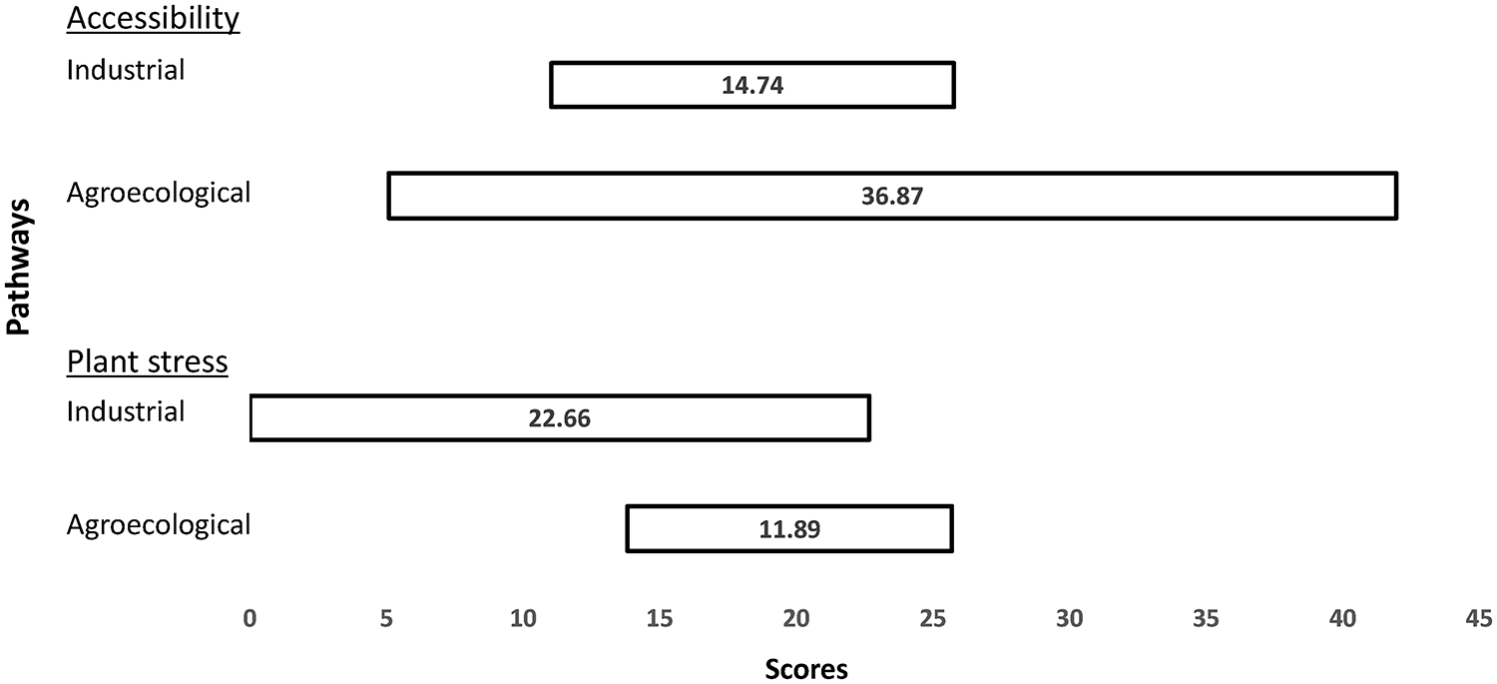
Extension workers’ perspective on Industrial and Agroecological pathways in Odisha, for the issues of accessibility and plant stress

Extension work these days is not just an activity of the government’s agricultural department. It is also carried out by non-governmental organisations. In close engagement with farmers, extension workers can support farmers’ adoption and adaptation of new STIs. Those extension workers who participated in the present study, focused on the issues of plant stress and accessibility. Their appraisals are shown in Fig. 2.

#### Accessibility

Under optimistic conditions, where farmers actively save seeds on farms and share them (and seed-saving knowledge) with each other, extension workers expect the agroecological pathway to perform better than the industrial pathway. However, their mean pessimistic score for the agroecological pathway is much lower than the corresponding score for the industrial pathway. This is because some extension workers noted the poor availability of traditional varieties, simply because in situ conservation efforts were not very common. Extension workers also highlighted that there was no policy support given to the agroecological pathway if farmers’ own efforts turn out to be ineffective. In contrast, for the industrial pathway, a significant amount of support from the government and the private sector means that extension workers consider its performance to be less uncertain.

#### Plant stress

For this issue, extension workers consider the industrial pathway to be associated with rather high uncertainty. While the industrial pathway has developed stress-tolerant and high yielding varieties (HYVs) of rice, extension workers argued that because new varieties were introduced every year or two, farmers were not able to learn about the performance of these varieties based on their own experience. In contrast, they considered many farmers as having substantial experience with the agroecological pathway’s traditional varieties, which is why this pathway was given higher optimistic and pessimistic scores.

### Researchers’ perspective

**Fig. 3:**
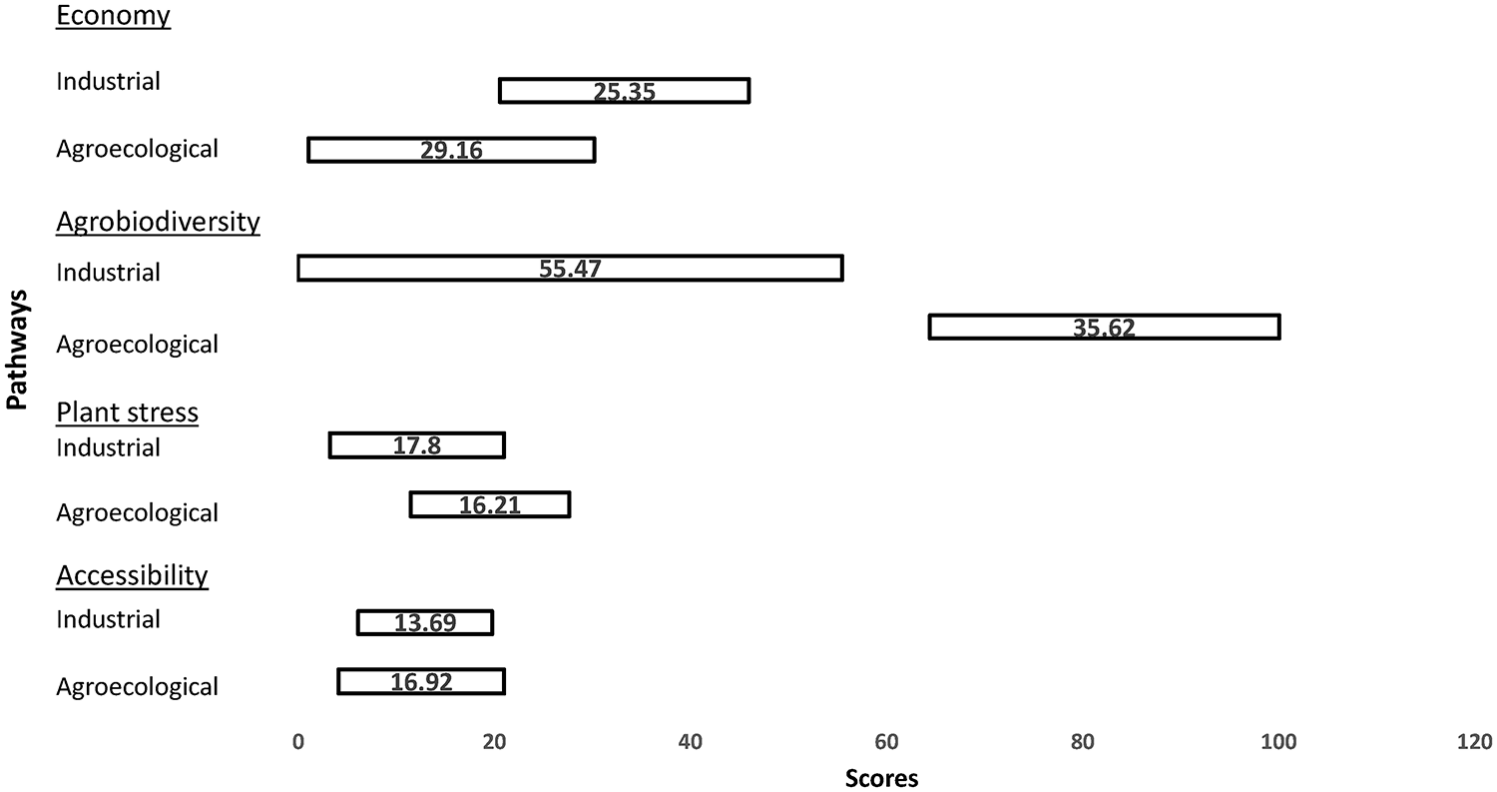
Researchers’ perspective on Industrial and Agroecological pathways in Odisha, for the issues of economy, agrobiodiversity, plant stress, and accessibility

Four issues are considered as salient by researchers. These are related to the economy, accessibility, agrobiodiversity and plant stress (see Fig. 3). Just like other perspectives, researchers’ view is ambiguous and complex. While the agroecological pathway is appraised as better performing for most issues, the industrial pathway is preferred for the economy.

#### Economy

Researchers appraised the industrial pathway with a higher mean optimistic score than the agroecological pathway, based on an expectation that laboratory-developed modern seeds generally yield higher than seeds conserved and developed by farmers. This expectation is a reflection of the researchers’ (most of whom are agricultural scientists) faith in the technical modifications they themselves engineer. As one scientist observes:

“In case of the new breeding strategies, we have…ourselves improved the [high-yielding] quality. We can’t change the quality of conserved materials.”

Consistent with the ambiguity of all perspectives, the researchers’ perspective was not homogeneous on the economy issue. There were some researchers who positively appraised the potential of ‘traditional’ seeds (or ‘landraces’) to yield economic benefits for farmers:

“Some landraces have excellent grain quality due to which they are much in demand in the market.”

“The yield will be almost the same as the landraces are very much adapted to the particular area. They are resistant to stress due to high-tolerance genes.”

#### Agrobiodiversity

 It is for this issue that ambiguity seems to have been rather marginal, with researchers appraising the agroecological pathway as overwhelmingly better performing. As shown in Fig. 3, the agroecological pathway was given a higher mean *pessimistic* score than the industrial pathway’s mean *optimistic* score. The latter pathway is also associated with a much higher uncertainty than the agroecological pathway. According to the researchers, the modern industrial pathway has a narrower genetic base than what the farmers and their partners have achieved in the agroecological pathway. They also highlight how the industrial pathway requires excessive chemical inputs, which adversely affect soil health and agricultural biodiversity.

#### Plant stress

While researchers consider the agroecological pathway’s seeds to be more tolerant towards stress in different small-scale local environments (as indicated by the pathway’s higher mean optimistic score), they believe that similar performance cannot be expected at larger scales. Under the latter conditions, they appraise the industrial pathway favourably and express appreciation for modern scientists’ efforts in gene selection for the development of stress-tolerant varieties in laboratories. These descriptions of different conditions by the researchers, can also explain the overlap observed in the two pathways’ scores for plant stress, as shown in Fig. 3.

#### Accessibility

The two pathways are appraised as rather similar in performance for the issue of accessibility. Researchers identify problems with both pathways when it comes to seed availability. For instance one researcher notes that, for the industrial pathway’s high-yielding varities, “the reach is still very poor especially for the new stress-tolerant varieties”. Local availability of the ‘Indigenous’ varieties (or ‘landraces’) developed in the agroecological pathway is considered to be better. However, due to the lack of institutional support for the agroecological pathway, researchers feel that the knowledge to develop new varieties by farmers (based on existing heritage and heirloom varieties) is widely lacking. As one researcher noted:

“There is no institutional mechanism to create awareness… about ongoing in situ conservation efforts [in the agroecological pathway]. The pathway has little support from the research community and there is very limited funding for civil society organizations trying to strengthen conservation efforts.”

### Policymakers’ perspective

**Fig. 4:**
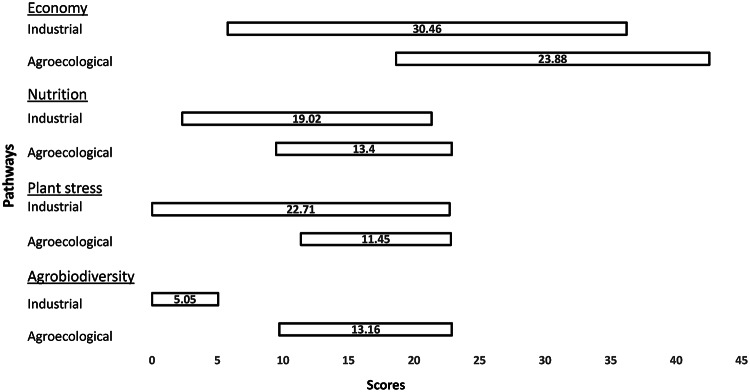
Policymakers’ perspective on Industrial and agroecological pathways in Odisha, for the issues of economy, agrobiodiversity, plant stress, and accessibility

Four issues are salient for the policymakers: agrobiodiversity, economy, nutrition, and plant stress. Their appraisals are of the two pathways are shown in Fig. 4.

#### Agrobiodiversity

Policymakers’ view is consistent with that of the researchers for agrobiodiversity: unsurprisingly the agroecological pathway is expected to perform much better than the industrial pathway. However, policymakers differ from researchers in terms of the uncertainties associated with their agrobiodiversity scores. For policymakers, the uncertainty associated with the agroecological pathway is very small in comparison with that of the industrial pathway. The latter pathway appears to be worse performing under all conditions.

#### Other issues

For the three other issues that policymakers consider as salient: plant stress, nutrition, and the economy, they appraise the industrial pathway as somewhat worse performing than the agroecological pathway, under both optimistic and pessimistic conditions. The agroecological pathway’s performance for these three issues is also appraised with lower uncertainties. However, for each of the three issues and unlike the issue of agrobiodiversity, the policymakers’ scores for the two pathways’ performance are overlapping. They explained the overlaps by raising concerns such as the following, with both pathways. For example:

“Monocropping [in industrial pathway] results in incidence of pests and diseases, which reduces the yield.”

“Farmers are cultivating landraces only in marginal lands and they use very little inputs and they are not getting good yields … Crop management practices and seed quality (in terms of purity) are poor.”

### Plural perspectives and implications

For the issue of agrobiodiversity, all MCM participants are broadly in agreement that the agroecological pathway performs better than the industrial pathway. This is not surprising given the definition of the agroecological pathway. Agrobiodiversity is related to sustainable development goals (SDGs) 15, 13, 3 and 2. For the issue of usability that pertains to SDGS 12 and 3, there seems to be a similar consensus in favour of the agroecological pathway. For all other issues except the economy, there is agreement among all participants’ plural perspectives that the agroecological pathway performs better than the industrial pathway under optimistic conditions (and often under pessimistic conditions too). Despite this clear picture in favour of the agroecological pathway, uncertainties and ambiguities expressed in the appraisals are pervasive, which means that the participants’ scores of the two pathways often overlap.

On the basis of our study conducted with just 20 participants, it is difficult to derive concrete policy implications that are widely applicable. By no means is our multi-criteria mapping, complete or ‘representative’. Yet our MCM results do help to illuminate and compare diverse pathways through a pluralist egalitarian lens that does not privilege a particular perspective. In the foregoing analysis thus we have focused on highlighting divergence between the four perspectives, alongside areas of convergence. For the latter, further research is needed to confirm what our comparative results indicate: that the agroecological pathway is better performing for realising the SDGs focused on agrobiodiversity (15, 13, 3 and 2) and usability (12 and 3). For other issues, the picture revealed by our MCM study is less straightforward in favour of a single pathway, as there are substantial overlaps between the two pathways’ performance scores. We interpret these overlaps to indicate that policy support cannot concentrate on just one pathway as is currently the case and has been in recent history (at least since the start of the Indian green revolution in the 1950s). It is crucial for sustainability to go beyond the ongoing institutional dominance of the modern industrial pathway, and redress historical neglect of the agroecological pathway based in Odisha’s Adivasi communities and their allies.

In general across India, the agroecological pathway in which Adivasi agricultural practices are developed is widely marginalised. Agrarian practices of Adivasi peoples living in or close to conservation zones have been vilified as ‘encroachment’ by government forest departments, which can lead to the expulsion of Adivasi farmers from their lands and forests (eg: Kapoor 2012; Navlakha 2012). Historically, shifting cultivation practices of many Adivasi communities were made much harder by the (post)colonial state’s control over forests and grazing areas that they classified as ‘waste lands’ (Guha 2006; de Hoop and Arora 2017). Similarly, many nomadic Adivasi peoples have been systematically targeted through hereditary criminalisation since 1871, when the first Criminal Tribes (and Castes) Act (CTA) was enacted by the British colonial state (Arora 2014). While the CTA was abolished after India’s independence in 1947, it was replaced in 1952 by the ‘Habitual Offenders Act’ that contained all provisions of the CTA except labelling targeted communities as ‘criminals by birth’ (Devy 2000). Such forms of interconnected injustices heaped on Adivasi peoples’ lifeways mean that their agricultural practices have not only been difficult to sustain (as peoples’ lands and other resources continue to be dispossessed for modern development projects). They have also been generally excluded from discussions of agricultural sustainability in India. These discussions reflect the dominance of the industrial agricultural pathway, particularly in policy circles (as discussed above in the sections on Literature Review, and Diverse STI pathways).

To address this widespread marginalisation, prioritisation of Adivasi agroecologies does imply shifting some subsidies and other funds out of the industrial pathway (to rebalance with the agroecological pathway). Adivasi smallholders and their partners in agroecological pathway also need to be *recognised* as ‘autonomous’ producers of crucial agricultural innovations for meeting the sustainable development goals. This recognition means many things, including protections against corporate exploitation of farmers’ freely shared knowledges (e.g., through restrictions on patenting of agroecological pathway’s seeds as well as their germplasm); political autonomy of Adivasi communities so that they can effectively struggle against the appropriation of their smallholdings and surrounding ecologies by larger farmers or by modern industry (often in the name of national, regional or local development); and supporting (Adivasi) smallholders to constitute and strengthen cooperatives for seeds and for other agricultural goods, through which the smallholders can sell in local markets if they so wish.

## Discussion and conclusions

It is evident from many of the perspectives in our MCM study and from India’s wider agricultural landscape that modern industrial pathways of STI development are currently dominant. Involving technologies like hybrid and genetically modified seeds, synthetic fertilizers and chemical pesticides (often developed in modern scientific laboratories), they attract a bulk of public and private resources and recognition. They are also often celebrated for heroic achievements, particularly of increasing crop yields since the so-called green revolution (GR) (Cabral et al. 2021). From adverse impacts on biodiversity and climate to ill health engendered by toxic agrochemicals, the many eco-social harms associated with industrial pathways’ dominance are often easily suppressed (Vasavi 2012). It is therefore crucial that alongside highlighting the sustainability transformations required in the dominant industrial pathways for agricultural development (Arora et al. 2020), equal consideration is given to the performance of alternative neglected STI pathways for a whole range of sustainability issues.

In this context, our research underscores the importance of illuminating diverse agricultural pathways through documentation and comparative analyses, despite our study’s limitation of defining and appraising just two alternative pathways. Through such illumination, it is possible to highlight which neglected STI pathway(s) can be prioritised – and how dominant STI pathways must be transformed – to address sustainability challenges (Ciarli et al. 2022). Central to transforming dominant STI pathways is agricultural research funding, which must broaden out from ruling specialisations in modern sciences and technologies such as biotechnology and agronomy (eg: Sumberg et al. 2013). Crucial in such broadening out might be participatory policy- and decision-making processes to identify and implement STI funding priorities: Participatory processes that privilege the perspectives of marginalised smallholders and their civil society allies amidst those of rural elites, agricultural experts, private corporations and public policymakers.

Foregrounding such plurality, albeit with small groups of participants, our study embraces the uncertainties associated with each perspective and the ambiguities of the picture yielded collectively by the perspectives. We illustrate how the uncertain perspectives converge and diverge from each other. For biodiversity and cultural appropriateness, all perspectives make the case for the agroecological pathway as better performing than the industrial pathway for developing rice seeds (ostensibly for climate resilience).^1^ While the collective picture was more divergent and ambiguous for other challenges underpinning the Sustainable Development Goals (SDGs), the plural perspectives indicate the need of nurturing the potentially significant alignment of the agroecological pathway to many SDGs. This means that neglected actors, practices and relations doing in situ conservation and development of rice seeds, particularly in communities of Indigenous (Adivasi in India) smallholders, must be recognised as central to sustainability if only to guarantee their autonomy over their lands and ecologies.

Such recognitions and guarantees are difficult to take hold without transforming dominant STI pathways constituted by wider hegemony of agricultural modernisation, so that they can coexist with alternative pathways associated with knowings of Indigenous and other vernacular smallholders. For such transformations, two conventionally modern assumptions need to be discarded for agricultural sustainability in India and elsewhere.

According to the first assumption, alternative (agroecological) pathways have lower crop yields and are therefore problematic from the perspective of food security (Patel 2021). Supporting alternative pathways is therefore assumed to undermine food security (and livelihoods) enabled by the industrial agriculture pathway (Jansen 2015). Discarding this assumption involves the transformation of asymmetric power relations, due to which modern industrial pathways are nurtured at the expense of agroecological pathways. Such a trransformation would mean that land, water and other socio-ecological bases of agroecological pathways are restituted and protected (through public policy and wider political struggles). Hopes may then be cherished for diverse pathways to equally thrive at the same time, rather than the weaker agroecological pathways becoming re-constituted or devoured by modern capitalisms and nationalisms (Ramdas and Pimbert 2024; Fitzpatrick et al. 2022).

The second modern assumption takes biodiversity losses (and other eco-social problems) as legitimate prices to pay for higher crop yields delivered by agricultural modernisation (see eg: Arora and van Dyck 2021; and Cabral et al. 2021 on GR narratives). By discarding this assumption, it may become possible to observe and imagine different relations between yield increases and biodiversity, while taking seriously issues such as cultural uses, nutrition and climate stresses. Yields – not just for an acre of land but also per unit of water usage – may then be considered across a wide diversity of (marginalised) ‘traditional’ crops such as millets and ‘agroecological’ rice varieties rather than continuing to foreground yields in one or two promoted staples and varieties bred by modern science (cf. Meek 2022).

Much of the foregoing requires the strengthening of social movements struggling for political autonomy of Adivasi (and other Indigenous) communities. Such movements are crucial for sustaining genuinely diverse alternative pathways grounded in multiple ways of knowing and being, in order to undo historical injustices and go beyond modern techno-universalisms that are prevalent in agricultural sustainability discussions today. It is the flourishing of Indigenous and vernacular lifeways that is thus required for diverse STI pathways to thrive and for agricultural sustainability goals to be realised.

## Data Availability

Anonymised data used in this article are available by writing to the corresponding author.

## Footnotes

1 While this result may be unsurprising for both cultural appropriateness and biodiversity (given their prominence in most agroecology pathways), it is still a crucial result that we must attend to, not least because of dramatic reductions observed in agrobiodiversity over the last few decades during which industrial agriculture has been promoted (see section titled **Diverse STI pathways for rice seeds in Odisha** above).
